# Enhancing the Kinetics of Vapor-based Polymerization by Pulsed Filament Approach

**DOI:** 10.1021/acs.langmuir.4c01172

**Published:** 2024-07-16

**Authors:** Jie Guo, Ranjita K. Bose

**Affiliations:** Department of Chemical Engineering, Product Technology, University of Groningen, Nijenborgh 4 Groningen AG 9747, the Netherlands

## Abstract

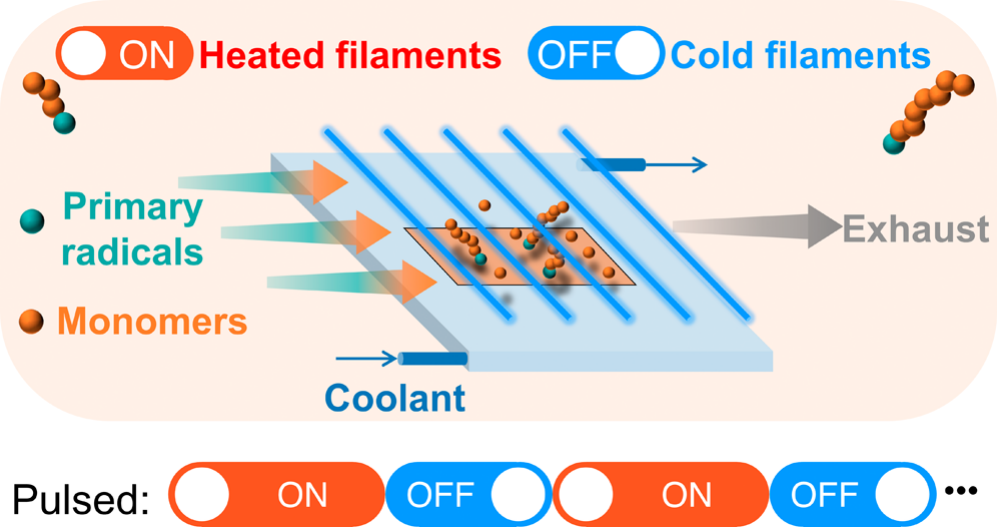

Initiated chemical vapor deposition is a versatile technique for synthesizing conformal polymer films on both planar and porous surfaces. It can retain functional groups and avoid undesired cross-linking. However, there is still room for enhancing its performance without altering the feed parameters. Here, we investigate a pulsed iCVD approach to improve the deposition process, achieved by switching on and off the resistively heated filament periodically. By strategically switching off the filament, a shortage of thermally activated primary radicals was created, which allowed uninterrupted chain propagation with fewer termination reactions and potentially increased monomer conversion rates. This has caused significantly faster deposition kinetics with a higher molecular weight and longer chain length for poly(glycidyl methacrylate) compared to continuous deposition. Spectra analyses confirmed that the functionality and stoichiometry ratios remained intact throughout the pulsed deposition process. The pulsed iCVD method is therefore a competitive and sustainable tool, demonstrating fast deposition kinetics and a well-preserved functionality.

## 1 Introduction

Vapor-based polymerization has been a competitive all-dry method to synthesize polymer films compared to conventional methods using solvents like spin coating,^1,2^ spray coating,^3^ and dip coating.^4^ Notably, it has the advantage of being able to produce uniform coatings not only on planar objects but also on nonplanar 3D substrates.^5^ Among various vapor-based techniques, initiated chemical vapor deposition (iCVD) can achieve high preservation of functional groups and significantly avoid cross-linking compared to plasma polymerization.^6,7^ ICVD is a continuous free radical polymerization process that typically involves a thermally decomposable initiator and vinyl monomer to deposit thin polymer films on different substrates.^8^ An array of filaments at 200–400 °C suspended above the substrates thermally activates initiators into primary radicals.^9^ These, along with monomers, diffuse and adsorb onto the cold surface below where the polymerization happens. Many commercially available vinyl monomers can be polymerized through iCVD, such as acrylates and methacrylates,^7,10−13^ fluorocarbon monomers,^14^ siloxane ring monomers,^15^ and cyclic monomers.^16^ The deposited films can strongly bond with certain polymeric substrates provided with a high surface energy.^17^ Moreover, it has the possibility to employ a “grafting from” approach through surface pretreatments with immobilized functional groups.^16^

The traditional iCVD technique often has a deposition rate of around 10–100 nm/min,^18^ which can be further improved with some adjustments. Given the fact that iCVD is a continuous process where the exhaust gas is often emitted out without recycling,^19^ it poses a sustainability concern due to low conversion rates of vapor precursors and potential waste generation. Therefore, it is important to fully utilize the vapors during the deposition and reduce the energy consumption to make the process more sustainable. Lau et al.^12^ found that high *P*_M_/*P*_M,sat_ (the ratio between monomer partial pressure and monomer saturation pressure at the cold surface) can result in a fast deposition rate. If we can increase *P*_M_/*P*_M,sat_ without altering the vapor feed rates, it would lead to an improved deposition rate and a higher polymer yield.

Bose et al.^13^ first reported a pulsed iCVD approach to obtain thicker films than continuous deposition. It was realized by switching on and off the resistively heated filaments in a periodic manner with all other parameters remained constant. During the filament “off” period, the substrate temperature would be slightly lower (5 °C) without heat radiation. Consequently, the saturation pressure of the monomer may decrease and result in more monomer availability at the surface compared with the “on” period. Since iCVD is an adsorption-limited process,^12^ this would naturally lead to a high *P*_M_/*P*_M,sat_ and fast deposition kinetics. For cyclohexyl methacrylate (CHMA), it is assumed that the lower molecular weight observed in pulsed iCVD may be a result of chain termination reactions that can still occur in the “off” period. However, this explanation does not fully elucidate the faster deposition kinetics with few initiators present. Further investigation is needed to understand the impact of the pulsed approach on the polymer molecular weight and chain length. Nevertheless, this pulsed approach holds practical promise for increasing monomer conversion rates and saving energy. In this study, we will analyze the continuous and pulsed deposition behavior of two different methacrylates, glycidyl methacrylate and furfuryl methacrylate. The effect of pulsed deposition on the polydispersity, number average molecular weight, and stoichiometry ratios will also be investigated to further understand the effect of pulsed deposition on polymer films.

## 2 Experiment

### 2.1 Initiated Chemical Vapor Deposition of Methacrylates

**Figure 1:**
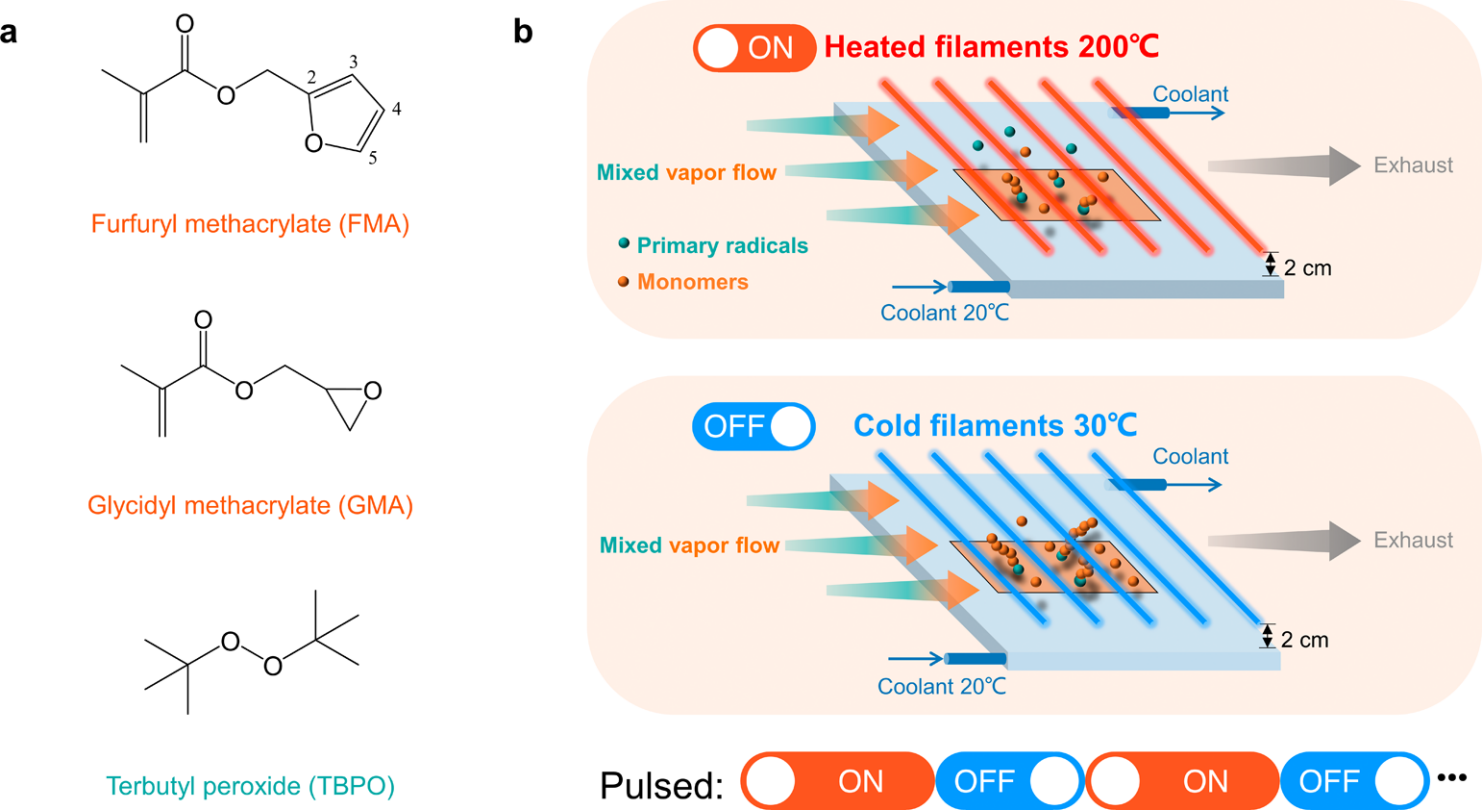
(a) Chemicals used in the vapor-based deposition, initiator: di-*tert*-butyl peroxide (TBPO), monomers: furfuryl methacrylate (FMA) and glycidyl methacrylate (GMA); (b) scheme of pulsed initiated chemical vapor deposition, the power supply connected to the filament is being switched on and off alternately, resulting different filament temperatures (*T*_F_) of 200 and 30 °C. The film deposition rate changed drastically from “on” mode to “off” mode and vice versa.

The initiated chemical vapor deposition (iCVD) of methacrylates takes place in a custom-built reaction chamber (Figure 1). Two monomers, glycidyl methacrylate (GMA, 97%, Sigma-Aldrich) and furfuryl methacrylate (FMA, 97%, Sigma-Aldrich), were used as received to prepare poly(glycidyl methacrylate) (pGMA) and poly(furfuryl methacrylate) (pFMA), respectively. Di-*tert*-butyl peroxide (TBPO, 98%, Sigma-Aldrich) was used as the thermal initiator. Monomers and initiators were heated to a certain temperature to deliver sufficient vapors into the chamber (GMA, 80 °C; FMA, 85 °C; TBPO, 25 °C). The gas lines were maintained at 110 °C to prevent possible condensations. The flow rates (*F*_M_, flow rate of monomer; *F*_I_, flow rate of initiator) were regulated by a metering valve and calibrated before each run. Inside the chamber, substrates (silicon wafer and aluminum) were positioned on a stage with circulating cooling liquid (ethylene glycol) to control the substrate temperature (*T*_s_). An array of stainless steel filaments (Goodfellow), situated 2 cm above the stage, was resistively heated to the required temperature using a DC power supply (Keysight) in order to generate enough primary initiator radicals. On the opposite side of the vapor inlets, the chamber was connected to a dry pump (Edwards) to reach vacuum. A throttling butterfly valve (MKS Instruments) between the chamber and pump, coupled with a Baratron capacitance manometer (MKS Instruments) monitoring the chamber pressure, allowed for precise pressure control. For a typical deposition, monomers and initiators were first vaporized and delivered into the chamber at constant flow rates. The initiators were then activated into radicals by the heated filaments. Both initiator radicals and monomer radicals were adsorbed onto the cold substrates, forming polymer chains and ultimately resulting in polymer films. In situ monitoring of film deposition on silicon substrates was achieved using a HeNe laser (633 nm, Thorlabs) through a transparent glass lid atop the chamber. The reflected beam of the film on silicon substrates was received by a photodetector. Since the laser signal intensity is a function of film thickness,^20^ interferometry was employed here to track changes in film thickness.

### 2.2 Pulsed Deposition

Pulsed initiated chemical vapor deposition is realized by switching on and off the power supply connected to the filament alternately to control the filament temperature. The schematic pulsed process is illustrated in Figure 1b. Initially, the power supply was switched on for the first 10 min, resembling continuous deposition and termed the “on” mode. Subsequently, it was switched off for the next 5 min, termed the “off” mode. During the “off” period, only the filament temperature (*T*_F_) changed without power input, while all other conditions remained unchanged compared to the “on” period, such as the flow rates of monomer and initiator, the chamber pressure (*P*_R_), and the temperature of cooling liquid. This cycle of two different modes was repeated alternately several times to obtain the pulsed polymer film. As shown in Figure 1b, *T*_F_ is 200 °C when the power supply is on and 30 °C when it is off. Table 1 shows the deposition parameters of pGMA for both continuous and pulsed depositions. The corresponding parameters of pFMA are detailed in Table S1.

**Table 1:** Deposition Parameters for pGMA^a^

pGMA iCVD parameters	P_R_/mTorr	F_M_/sccm	F_I_/sccm	T_S_/°C	T_F_/°C	P_M_/P_M,sat_	P_I_/P_I,sat_
continuous deposition	200	1.50	1.00	22	200	0.40	0.0033
	250	1.50	1.00	22	200	0.50	0.0041
	300	1.50	1.00	22	200	0.61	0.0049
	350	1.50	1.00	22	200	0.71	0.0057
	400	1.50	1.00	22	200	0.81	0.0065
pulsed deposition	300	1.50	1.00	22	200/30	0.61	0.0049

a *P*_R_, chamber pressure; *F*_M_, flow rate of monomer; *F*_I_, flow rate of initiator; *T*_S_, substrate temperature; *T*_F_, filament temperature; *P*_M_/*P*_M,sat_, ratio of monomer partial pressure to the saturated pressure of monomer at *T*_S_; and *P*_I_/*P*_I,sat_, ratio of initiator partial pressure to the saturated pressure of initiator at *T*_S_.

### 2.3 Characterizations

The thickness of the iCVD deposited polymer is characterized through the film on silicon wafers, serving as a reference for the film on other substrates. A profilometer (Bruker DektakXT) was used to measure the thickness in three different regions on each silicon wafer. Fourier transform infrared spectroscopy (FTIR, Shimadzu IRtracer-100) was employed to analyze the structure of the polymer film on silicon wafers, with a bare silicon wafer as the background. The spectra were recorded in absorbance mode from 500 to 4000 cm^–1^ with 64 scans and a resolution of 2 cm^–1^.

The number average molecular weight of the deposited polymer film was determined through the following procedures: first, the polymer film (an area of 75 cm^2^, with a thickness of approximately 1 μm) was dissolved from the substrates using tetrahydrofuran (THF). It should be noted that sonication cannot be used to dissolve the polymer film, as it would cleave the polymer chains into small fragments and thus result in a decreased molecular weight.^21^ The solution was then appropriately concentrated by evaporating the solvent at room temperature for further analysis. Gel permeation chromatography (GPC, with a Mixed-E column) was used to measure the number average molecular weight and the polydispersity of the polymer film with toluene as the internal standard. The results were processed and calculated based on a set of narrow polystyrene standards of known molecular weight and distribution. Additionally, ^1^H NMR spectra were recorded on a Varian Mercury Plus 400 MHz spectrometer using deuterated chloroform (CDCl_3_) as a solvent. The remaining aforementioned polymer film solution was further concentrated until all THF evaporated before adding CDCl_3_.

## 3 Results and Discussion

### 3.1 Pulsed Initiated Chemical Vapor Deposition Polymer Film

**Figure 2:**
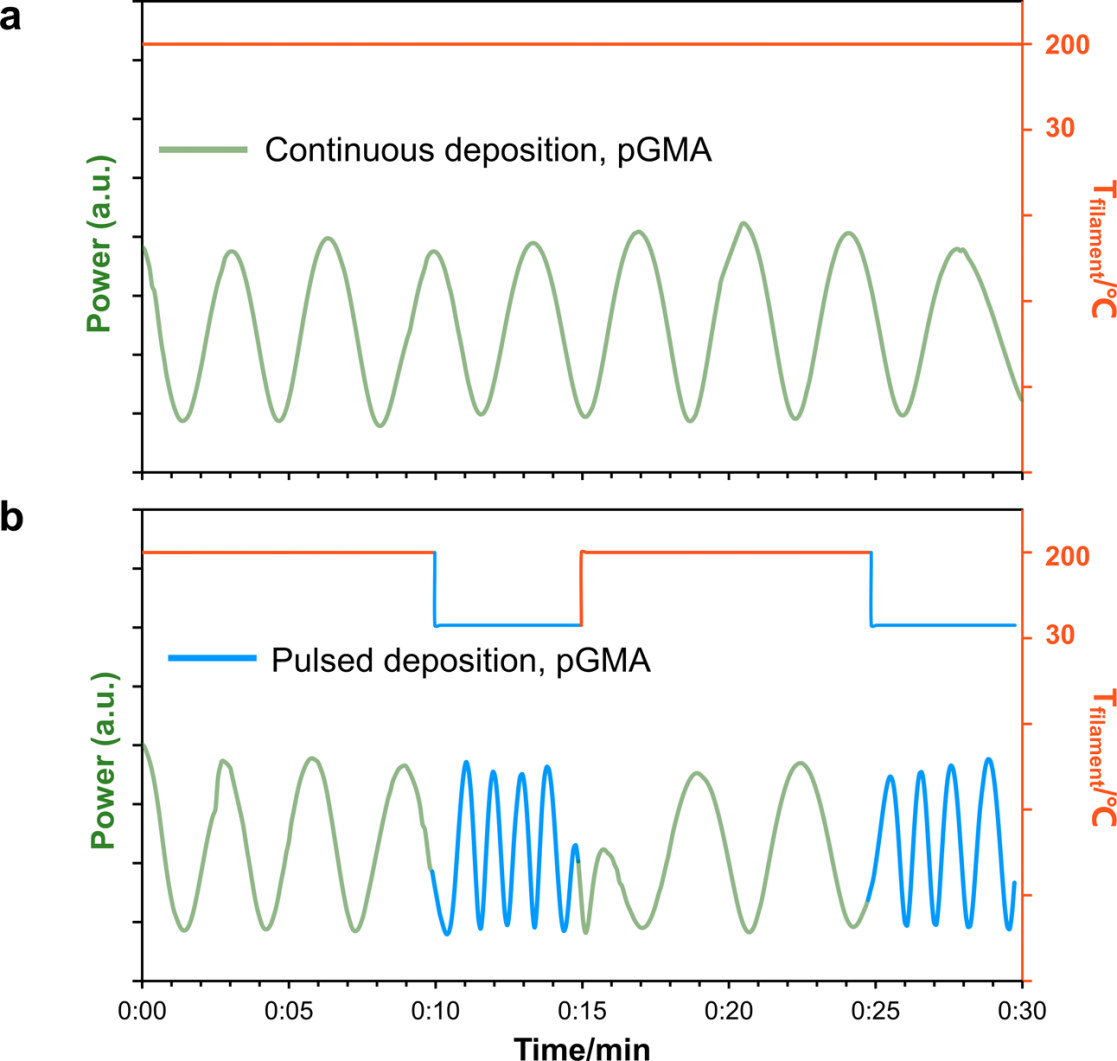
Laser interference pattern of pGMA films on silicon substrates during initiated chemical vapor deposition: (a) continuous deposition, light green; (b) pulsed deposition, blue (a.u., arbitrary units).

Two methacrylates with different functional groups, glycidyl groups and furan groups, were selected as the monomers here to investigate the difference between the pulsed iCVD process and continuous iCVD deposition. The in situ laser signal deposition data were first used to predict the deposition behavior of the two methacrylates. Figure 2 illustrates the laser interference pattern of the pGMA film during both continuous deposition and pulsed deposition. Typically, the laser signal intensity (power) exhibits a cosine relation as a function of time and is colored light green during continuous deposition. Here, we define one deposition cycle based on one cosine cycle of the interference pattern, which usually stands for a certain fixed thickness (around 100 nm). More cycles indicate a higher thickness of the deposited film.^20^ During continuous deposition, the time required to complete each cycle remained constant throughout the entire deposition process with *T*_F_ at a constant 200 °C. Conversely, in pulsed deposition, the time to complete each deposition cycle varied when *T*_F_ changed from 200 to 30 °C and vice versa. In the first 10 min of pulsed deposition, the laser signal behaved similarly to continuous deposition. However, after *T*_F_ dropped to 30 °C, the number of deposition cycles (in blue) increased about 220% during the 5 min “off” period compared to the parallel continuous 5 min “on” period. When the power supply was switched back on, the deposition returned to continuous mode in the next 10 min. Subsequently, as the filament was switched off again, the deposition cycles experienced the same increase. The laser interference pattern of pFMA is shown in Figure S2. In this case, no obvious changes in the number of deposition cycles were observed with the alteration of the *T*_F_. From this, we can infer that the pulsed filament approach brought higher deposition rates for pGMA but not for pFMA.

**Figure 3:**
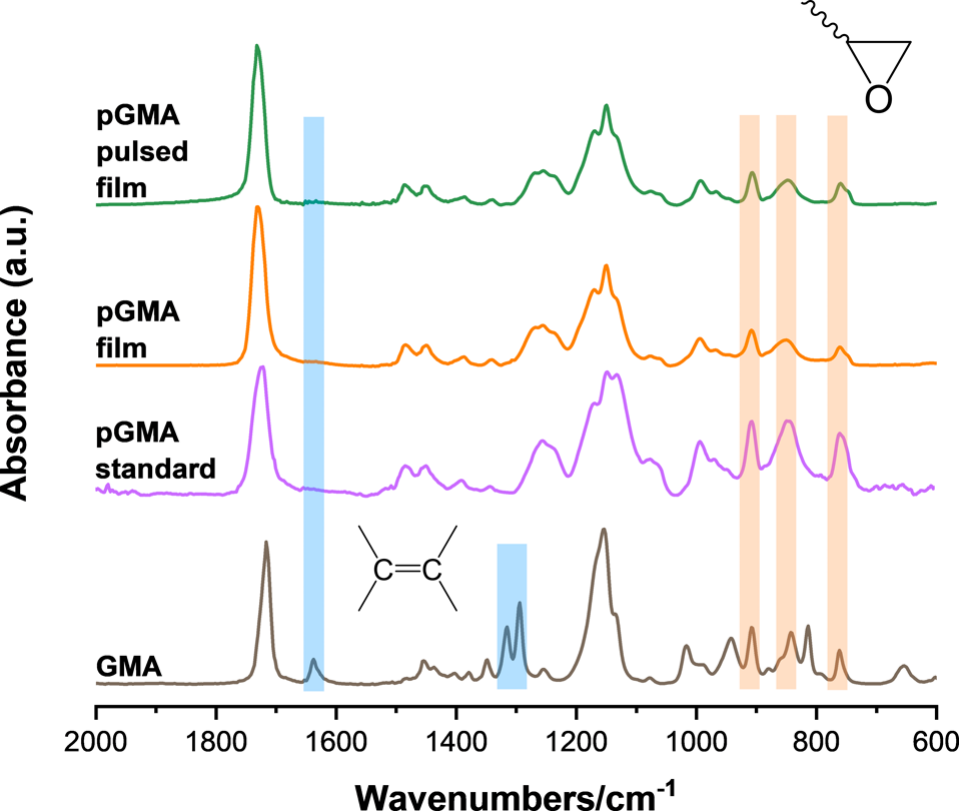
Infrared spectra of pGMA. The four curves represent three kinds of pGMA: pulsed iCVD pGMA film, continuous iCVD pGMA film, standard pGMA (Sigma-Aldrich), and the monomer GMA.

The infrared (IR) spectra of different pGMA films are shown in Figure 3. It is evident that the C=C stretching vibrations at 1637 cm^–1^ and two C–H in-plane deformations at 1315 and 1294 cm^–1^ all disappeared in three types of pGMA in comparison to the GMA monomer.^22^ The characteristic peaks of the epoxy groups were all preserved in two iCVD pGMA films (continuous and pulsed) at 908, 850, and 761 cm^–1^, respectively. This suggests that the functional groups remained intact after pulsed deposition.^7^ The pGMA film spectra also closely match that of standard pGMA (conventionally polymerized, Sigma-Aldrich), providing further confirmation of the successful iCVD polymerization of GMA. As for the IR spectra of different pFMA films (Figure S1), the absorbance peaks of the C=C double bond were exclusively present in the FMA monomer.^11^ The characteristic peaks of furan groups at 1502, 1227, and 1017 cm^–1^ were retained in both iCVD pFMA films.^23,24^ However, there is one additional weak shoulder at 1776 cm^–1^ in the iCVD pFMA film compared to that in the standard pFMA (synthesized by atom transfer radical polymerization). This discrepancy may be attributed to different types of carbonyl environments formed by allylic radicals from the 5′ position of the furan ring.^25,26^

**Figure 4:**
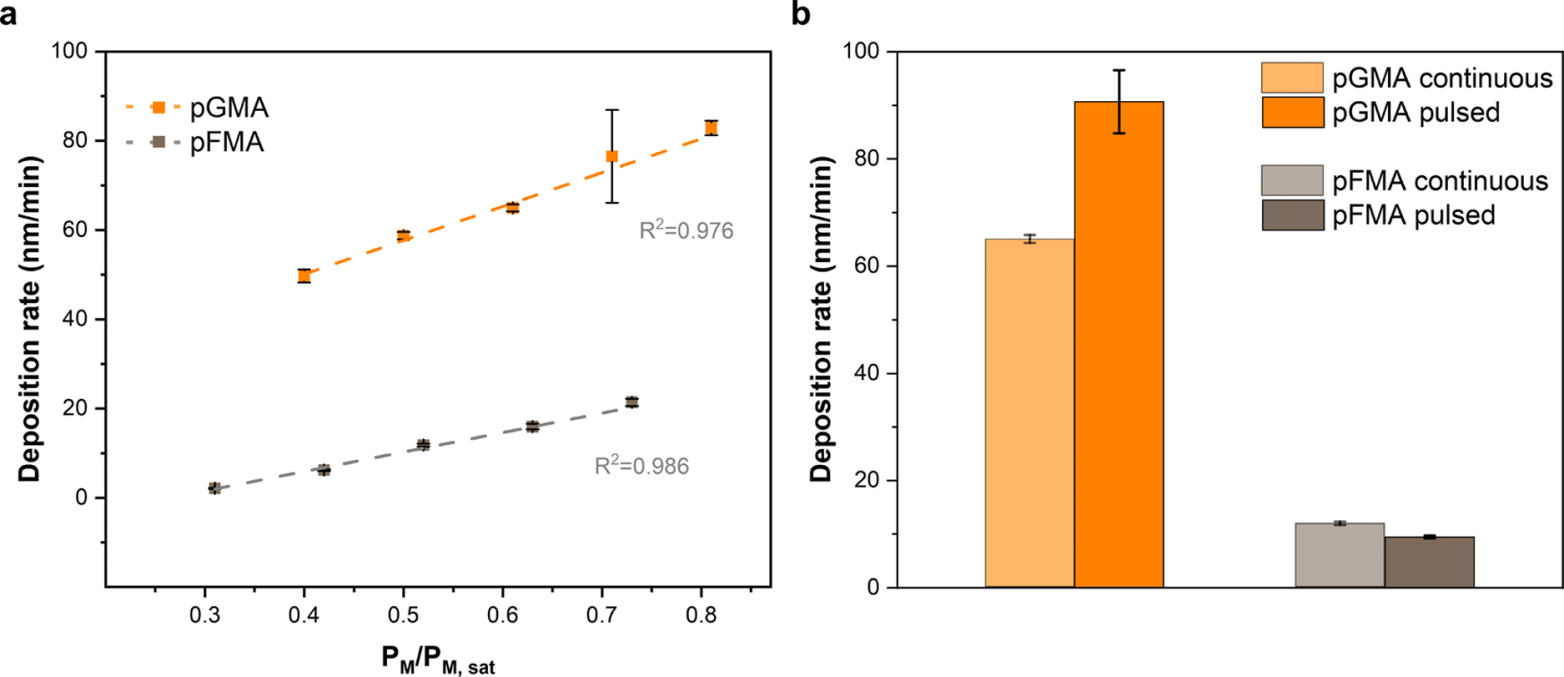
(a) Deposition rate curves of pGMA, and pFMA; (b) comparison of deposition rates between continuous and pulsed depositions under the same conditions for pGMA and pFMA, respectively.

Figure 4a depicts the continuous deposition rate curves of pGMA and pFMA within a similar P_M_/P_M,sat_ range of 0.3–0.8 (varied by changing the reactor pressure). Both exhibited a linear relationship with increasing monomer concentrations. However, it was observed that the deposition rate of pGMA was much higher with faster deposition kinetics compared with that of pFMA under the same conditions. The IR spectra of pGMA at different *P*_M_/*P*_M,sat_ values showed the same absorbance peaks, validating that there were no chemical changes in the films when varying the reactor pressure (same for pFMA). Subsequently, pulsed deposition was carried out at *P*_M_/*P*_M,sat_ = 0.61 for pGMA and 0.52 for pFMA, respectively. The deposition rate of pGMA increased by about 50% from 64.90 to 90.67 nm/min in the pulsed mode (Figure 4b). The pulsed deposition rate was even slightly higher than the one at *P*_M_/*P*_M,sat_ = 0.81 (82.90 nm/min), which makes it possible to reach much higher deposition kinetics based on the pulsed filament alone without changing any other feed conditions. Control tests were performed by switching off the filament throughout the entire deposition. No films were deposited with undetectable thickness, confirming the necessity of the “on” period to initiate polymerization. This could be particularly beneficial for monomers with slow kinetics even at a high *P*_M_/*P*_M,sat_ value^27,28^ or for low vapor pressure monomers where achieving a high *P*_M_/*P*_M,sat_ is not easily feasible.

**Figure 5:**
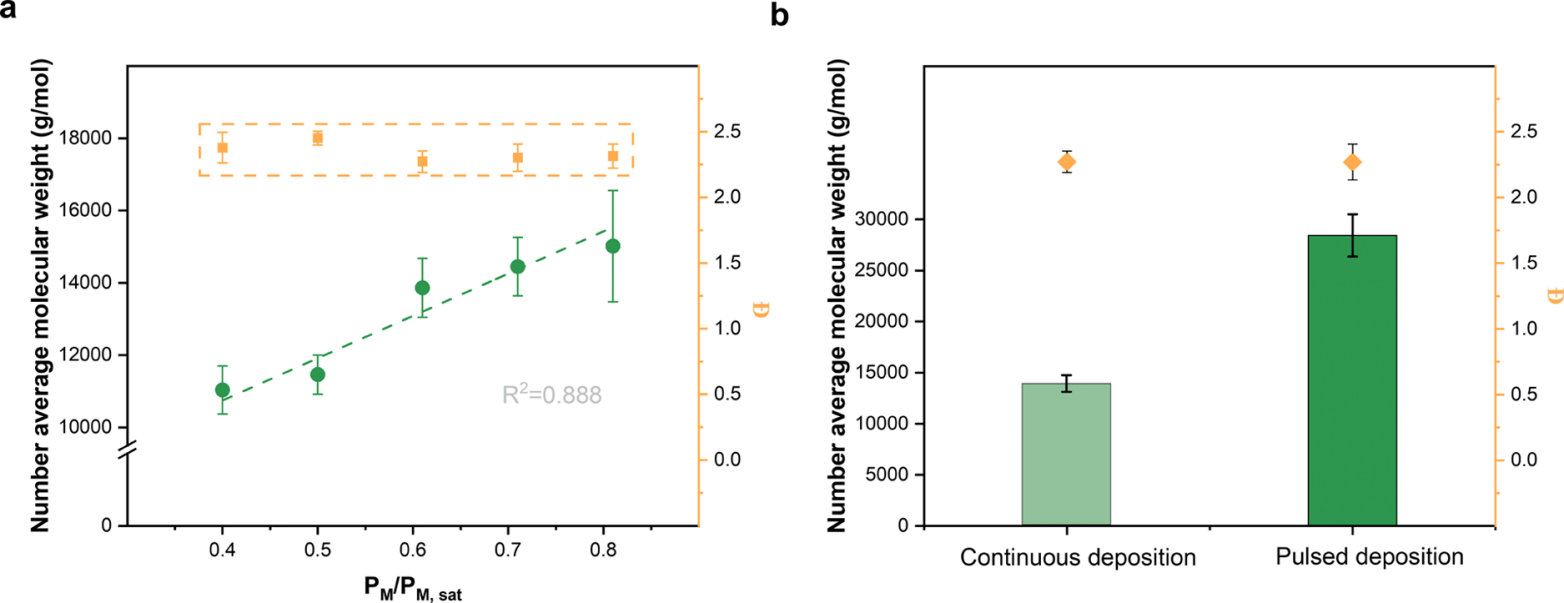
(a) Number average molecular weight (*M*_n_) of pGMA film under different *P*_M_/*P*_M,sat_ values; (b) comparison of *M*_n_ and polydispersity *Đ* between continuous and pulsed depositions.

When the monomer changed to FMA, no obvious differences in deposition rates were found between continuous and pulsed depositions (11.80 and 9.47 nm/min). It is possible that without the presence of initiator radicals, the slow kinetics of FMA iCVD deposition did not promote the chain propagation during the pulsed mode and resulted in less difference in deposition rates compared to continuous deposition. Combining with the low molecular weight of pulsed iCVD CHMA,^13^ it is speculated that the chain termination reactions for these two monomers were faster than the chain propagation, preventing polymer growth. This indicates that pulsed deposition may not be suitable for certain monomers depending on the molecular structure and the deposition kinetics. Therefore, only pGMA films were analyzed for the polydispersity and number average molecular weight *M*_n_ (Figure 5). The deposited pGMA molecular weights demonstrated a positive linear correlation with *P*_M_/*P*_M,sat_. Naturally, increasing monomer concentrations led to more chances of chain propagation and a higher *M*_n_. The *M*_n_ value ranging from 11036 to 15017 g/mol is in accordance with the reference at similar initiator/monomer ratios (0.6 reference, 0.67 this paper).^29^ The polydispersity of deposited pGMA remained around 2.34 throughout the aforementioned *P*_M_/*P*_M,sat_ range, indicating a continuous distribution of free radical polymerizations. Interestingly, the *M*_n_ increased nearly two times from 13861 g/mol in continuous deposition (*P*_M_/*P*_M,sat_ = 0.61) to 28430 g/mol in pulsed deposition. Moreover, this significant increase did not notably influence the polydispersity (2.26) as well, suggesting a consistent molecular mass distribution through the pulsed approach.

Combining all the findings presented above, it can be inferred that in the pulsed pGMA process, at first, the deposition mirrored the continuous deposition. Then during the filament “off” period, the initiator ceased to break into primary radicals due to the absence of thermal excitation. However, since the flow rates of monomer and initiator remained unchanged, the ratio between monomer radicals and initiator radicals rapidly increased with fewer initiator radicals generated. Therefore, the propagation step of polymerization likely continued to take place because of excess monomer radicals. It is also assumed that the rate of propagation exceeded the rate of termination with the decreasing numbers of initiators, leading to more propagation steps and the growth of a longer polymer chain with higher *M*_n_. As expected, this would bring a significantly higher deposition rate compared to the traditional continuous approach. Additionally, the higher *M*_n_ has further benefits of increased mechanical properties such as tensile strength, modulus,^30^ impact resistance,^31^ and creep resistance.^32^ These positive effects can make the deposited films more suitable for protective coatings, adhesives, and microelectronics.

Since the feed rates remained constant, it is reasonable to presume that the monomer conversion rates have increased with faster deposition rates. The exact value can be calculated by quantifying the concentration of monomer at the surface through vacuum-compatible quartz crystal microbalance^12^ (QCM) in future studies. Based on a preliminary study, at a time interval shorter than 5 min, the pulsed approach did not improve the deposition rates significantly. While at time intervals longer than 5 min, initiator radicals were gradually all consumed without new ones generated. It then led to a decreased deposition rate. Therefore, the time interval for the “off” period was set at 5 min to reach a balance between these two factors. However, the ratio between the “on” period and “off” period may be optimized to further improve the deposition rates and monomer conversion rates and increase the *M*_n_.

### 3.2 Stoichiometry Ratios of Pulsed pGMA Film

**Figure 6:**
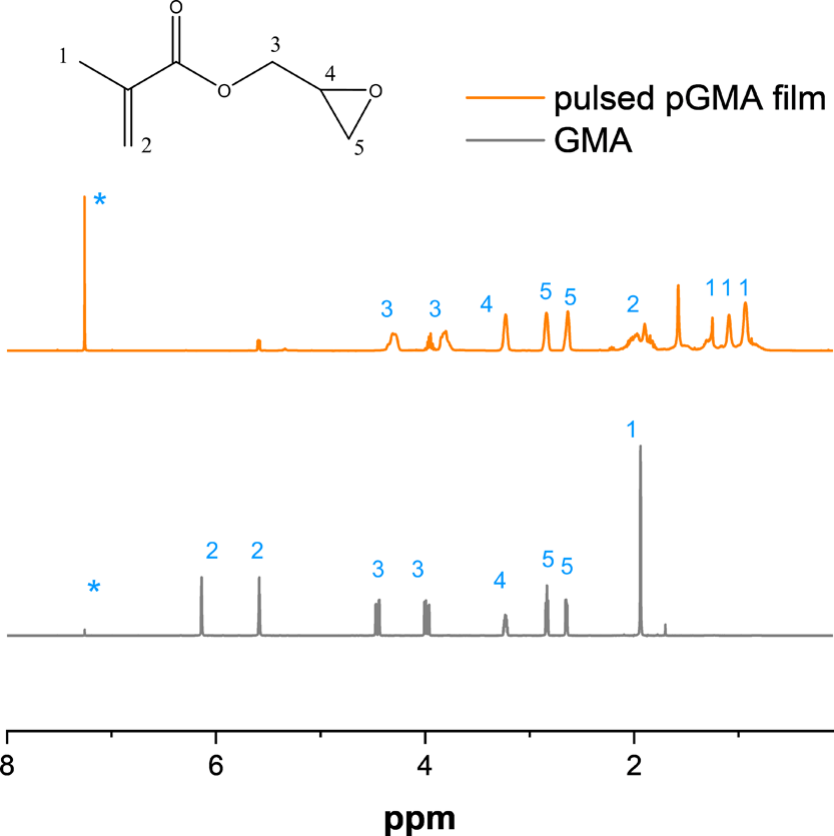
^1^H NMR spectra of the pulsed pGMA film and monomer GMA (*: CDCl_3_).

Figure 6 illustrates the ^1^H NMR spectra of the deposited pulsed pGMA film. The chemical shifts of different hydrogen atoms are in align with Mao et al.’s reported results of continuous deposited pGMA film.^29^ Epoxy functional groups at 2.64, 2.84, and 3.22 ppm were well preserved, while the vinyl groups at 5.60 and 6.14 ppm nearly disappeared in the film. There was a small signal at 5.60 ppm, which could be attributed to monomer adsorbed onto the cold surface from excessive monomer radicals. It correlates with the small C=C peak at 1502 cm^–1^ for pulsed pGMA film in Figure 3. The integrals of the corresponding hydrogens are listed in Table 2. The peak area ratio of different signals’ integrals for pGMA film is in good agreement with the monomer. The pulsed pGMA film successfully maintained the same stoichiometry ratios as the monomer and the linear polymeric structure as the continuous pGMA film. Therefore, this further validates that the pulsed approach had few or no effects on the film at the molecular structure level.

**Table 2:** Integrals of the Pulsed pGMA Film

			**area (a.u.)**	
^**1**^**H NMR**	**theoretical protons**	**chemical shifts (ppm)**	**GMA**	**pulsed pGMA film**
H_5_	2	2.84, 2.64	1.97	2.04
H_4_	1	3.22	0.90	0.99
H_3_	2	4.30, 3.82	2.01	2.00
H_2_	2	1.79–2.16	2.08	2.40
H_1_	3	0.92, 1.09,1.28	3.08	4.01

### 3.3 Areal Density of Deposited pGMA Films

To ascertain the precise number of functional groups of the deposited pGMA film, a semiquantitative GPC method previously reported by Bose et al.^16^ was employed in this study to estimate the absolute weight of deposited pGMA film. First, a series of standard pGMA solutions (*M*_n_ 10000–20000 g/mol, in THF) with different weight concentrations were used to make a standard calibration curve, correlating the GPC peak area with weight concentration (Figure S4). All pGMA films were easily dissolved from the substrates, indicating that both continuous and pulsed films were not cross-linked.^29^ Aluminum substrates were used since their good thermal conductivity ensured homogeneous surface temperatures, which was crucial for this study. By comparing the integrals of the GPC peak area, we estimated the weight concentration of continuous pGMA film solutions and further determined the mass areal density to be 123.6 μg/cm^2^. With an *M*_n_ of 13861 g/mol from GPC, the molar areal density was calculated as 8916.8 pmol/cm^2^, and the chain numbers were 53.7 chains/nm^2^. Similarly, we calculated the values for the pulsed pGMA film (Table 3). Both molar areal density (9966.5 pmol/cm^2^) and chain numbers (60.0 chains/nm^2^) were close to those of the continuous pGMA film. This data is significantly higher than the areal density of 0.75–1.5 chains/nm^2^ from conventional pGMA film reported by Liu et al.^33^ Due to the higher *M*_n_, the pulsed mass areal density was 283.4 μg/cm^2^, two times higher than continuous deposition. In another “graft from” study,^34^ the surface pretreated polyethylene had a GMA mass areal density as high as 650 μg/cm^2^. The surface pretreatments provided peroxide initiating groups for graft polymerization, which enabled easier attachment of the monomers onto the surface compared to the “graft-to” approach and thus resulted in a high mass areal density. Considering that no surface pretreatments were made in our study, the areal density of pulsed iCVD is still comparable. It may be further increased with different ratios between the “on” and “off” periods.

**Table 3:** Estimation of the Areal Density of pGMA Films

	continuous pGMA film	pulsed pGMA film	reference
mass areal density (μg/cm^2^)	123.6	283.4	650^34^
molar areal density (pmol/cm^2^)	8916.8	9966.5	
chains/nm^2^	53.7	60.0	0.75–1.5^33^

## 4 Conclusions

This work investigated the impact of the pulsed iCVD approach on polymer film deposition. A big advantage of the pulsed approach is that by switching off the filament shortly, the pGMA deposition rate can be raised significantly while preserving functional groups and keeping the stoichiometry ratios of linear polymer structure. The number average molecular weight and mass areal density of pulsed iCVD pGMA increased 2-fold in comparison to those of continuous iCVD pGMA. The enhancement observed can be attributed to increased chain propagations during the “off” period. The lower filament temperature led to a decrease in initiator radicals, subsequently slowing chain terminations. While monomers remained unaffected by the filament, existing chains were able to continue propagating, ultimately contributing to improved deposition rates. However, it is essential to note that the pulsed approach can be a monomer-dependent technique. Only GMA exhibited both rapid pulsed deposition kinetics and high molecular weight, whereas FMA did not show any improvement with the pulsed approach. Future research endeavors could explore a broader range of monomers for pulsed deposition. In addition, the pulsed conditions, such as the ratio between the “on” and “off” periods and the initiator flow, could be further fine-tuned to improve the film deposition. In summary, among various vapor-based techniques, the pulsed iCVD approach is a competitive tool capable of improving deposition kinetics and monomer conversion rates while reducing energy consumption.

## Supporting Information Available

(Figure S1) Infrared spectra of pFMA; (Figure S2) laser interference pattern of the pFMA film on silicon substrates; (Figure S3) infrared spectra of pGMA and pFMA at different *P*_M_/*P*_M,sat_ values; (Table S1) deposition parameters for pFMA; (Figure S4) standard calibration curve of the integrals of standard pGMA against weight percentage (PDF)

## References

[ref1] Norrman K.; Ghanbari-Siahkali A.; Larsen N. B. 6 Studies of spin-coated polymer films. Annu. Rep. Section C (Phys. Chem.) 2005, 101, 174 10.1039/b408857n.

[ref2] Soto-Cantu E.; Lokitz B. S.; Hinestrosa J. P.; Deodhar C.; Messman J. M.; Ankner J. F.; Kilbey S. M. II Versatility of Alkyne-Modified Poly(Glycidyl Methacrylate) Layers for Click Reactions. Langmuir 2011, 27 (10), 5986–5996. 10.1021/la2000798. 21506527

[ref3] Lau K. K. S.; Gleason K. K. Applying HWCVD to particle coatings and modeling the deposition mechanism. Thin Solid Films 2008, 516 (5), 674–677. 10.1016/j.tsf.2007.06.045.

[ref4] Roland S.; Gamys C. G.; Grosrenaud J.; Boissé S.; Pellerin C.; Prud’homme R. E.; Bazuin C. G. Solvent Influence on Thickness, Composition, and Morphology Variation with Dip-Coating Rate in Supramolecular PS-b-P4VP Thin Films. Macromolecules 2015, 48 (14), 4823–4834. 10.1021/acs.macromol.5b00847.

[ref5] Baxamusa S. H.; Im S. G.; Gleason K. K. Initiated and oxidative chemical vapor deposition: a scalable method for conformal and functional polymer films on real substrates. Phys. Chem. Chem. Phys. 2009, 11 (26), 5227 10.1039/b900455f. 19551189

[ref6] Friedrich J. Mechanisms of Plasma Polymerization – Reviewed from a Chemical Point of View. Plasma Processes and Polymers 2011, 8 (9), 783–802. 10.1002/ppap.201100038.

[ref7] Mao Y.; Gleason K. K. Hot Filament Chemical Vapor Deposition of Poly(glycidyl methacrylate) Thin Films Using tert-Butyl Peroxide as an Initiator. Langmuir 2004, 20 (6), 2484–2488. 10.1021/la0359427. 15835714

[ref8] Gleason K. K. Designing Organic and Hybrid Surfaces and Devices with Initiated Chemical Vapor Deposition (iCVD). Adv. Mater. 2024, 36 (8), 2306665 10.1002/adma.202306665. 37738605

[ref9] Tenhaeff W. E.; Gleason K. K. Initiated and Oxidative Chemical Vapor Deposition of Polymeric Thin Films: iCVD and oCVD. Adv. Funct. Mater. 2008, 18 (7), 979–992. 10.1002/adfm.200701479.

[ref10] Chan K.; Gleason K. K. Initiated CVD of Poly(methyl methacrylate) Thin Films. Chem. Vap. Deposition 2005, 11 (10), 437–443. 10.1002/cvde.200506381.

[ref11] Chen G.; Gupta M.; Chan K.; Gleason K. K. Initiated Chemical Vapor Deposition of Poly(furfuryl methacrylate). Macromol. Rapid Commun. 2007, 28 (23), 2205–2209. 10.1002/marc.200700466.

[ref12] Lau K. K. S.; Gleason K. K. Initiated Chemical Vapor Deposition (iCVD) of Poly(alkyl acrylates): An Experimental Study. Macromolecules 2006, 39 (10), 3688–3694. 10.1021/ma0601619.

[ref13] Bose R. K.; Heming A. M.; Lau K. K. S. Microencapsulation of a Crop Protection Compound by Initiated Chemical Vapor Deposition. Macromol. Rapid Commun. 2012, 33 (16), 1375–1380. 10.1002/marc.201200214. 22573697

[ref14] Laird E. D.; Bose R. K.; Wang W.; Lau K. K. S.; Li C. Y. Carbon Nanotube-Directed Polytetrafluoroethylene Crystal Growth via Initiated Chemical Vapor Deposition. Macromol. Rapid Commun. 2013, 34 (3), 251–256. 10.1002/marc.201200678. 23225149

[ref15] Coclite A. M.; Ozaydin-Ince G.; d’Agostino R.; Gleason K. K. Flexible Cross-Linked Organosilicon Thin Films by Initiated Chemical Vapor Deposition. Macromolecules 2009, 42 (21), 8138–8145. 10.1021/ma901431m.

[ref16] Bose R. K.; Nejati S.; Stufflet D. R.; Lau K. K. S. Graft Polymerization of Anti-Fouling PEO Surfaces by Liquid-Free Initiated Chemical Vapor Deposition. Macromolecules 2012, 45 (17), 6915–6922. 10.1021/ma301234z.

[ref17] Im S. G.; Bong K. W.; Lee C.-H.; Doyle P. S.; Gleason K. K. A conformal nano-adhesive via initiated chemical vapor deposition for microfluidic devices. Lab Chip 2009, 9 (3), 411–416. 10.1039/B812121D. 19156290

[ref18] Gleason K. K. CVD polymers: fabrication of organic surfaces and devices; John Wiley & Sons, 2015.

[ref19] Movsesian N.; Dianat G.; Gupta M. Downstream Monomer Capture and Polymerization during Vapor Phase Fabrication of Porous Membranes. Ind. Eng. Chem. Res. 2019, 58 (23), 9908–9914. 10.1021/acs.iecr.9b01315.

[ref20] Cruden B.; Chu K.; Gleason K.; Sawin H. Thermal Decomposition of Low Dielectric Constant Pulsed Plasma Fluorocarbon Films: I. Effect of Precursors and Substrate Temperature. J. Electrochem. Soc. 1999, 146 (12), 4590 10.1149/1.1392679.

[ref21] Akyüz A.; Catalgil-Giz H.; Giz A. T. Kinetics of Ultrasonic Polymer Degradation: Comparison of Theoretical Models with On-Line Data. Macromol. Chem. Phys. 2008, 209 (8), 801–809. 10.1002/macp.200700533.

[ref22] Mistry B. A Handbook of spectroscopic data, chemistry (UV, IR, PNMR, CNMR and Mass Spektoscopy); BKM Science College; 2009.

[ref23] Tarducci C.; Badyal J. P. S.; Brewer S. A.; Willis C. Diels–Alder chemistry at furan ring functionalized solid surfaces. Chem. Commun. 2005, 3, 406–408. 10.1039/B412906G. 15645053

[ref24] Kavitha A. A.; Choudhury A.; Singha N. K. Controlled Radical Polymerization of Furfuryl Methacrylate. Macromol. Symp. 2006, 240 (1), 232–237. 10.1002/masy.200650828.

[ref25] Lange J.; Rieumont J.; Davidenko N.; Sastre R. Kinetics modelling of the crosslinking in the photopolymerization of furfuryl methacrylate in bulk. Comput. Theor. Polym. Sci. 1999, 9 (1), 63–72. 10.1016/S1089-3156(99)00004-5.

[ref26] Lange J.; Davidenko N.; Rieumont J.; Sastre R. Study of network formation in furfuryl methacrylate photopolymerisation at different temperatures. The Tobita method applied to the polymerisation at low conversions. Polymer 2002, 43 (3), 1003–1011. 10.1016/S0032-3861(01)00619-X.

[ref27] Khlyustova A.; Yang R. Initiated Chemical Vapor Deposition Kinetics of Poly(4-aminostyrene). Frontiers in Bioengineering and Biotechnology 2021, 9, 670541 10.3389/fbioe.2021.670541. 33937221 PMC8085358

[ref28] Chan K.; Gleason K. K. A Mechanistic Study of Initiated Chemical Vapor Deposition of Polymers: Analyses of Deposition Rate and Molecular Weight. Macromolecules 2006, 39 (11), 3890–3894. 10.1021/ma051776t.

[ref29] Mao Y.; Felix N. M.; Nguyen P. T.; Ober C. K.; Gleason K. K. Towards all-dry lithography: Electron-beam patternable poly(glycidyl methacrylate) thin films from hot filament chemical vapor deposition. Journal of Vacuum Science & Technology B: Microelectronics and Nanometer Structures Processing, Measurement, and Phenomena 2004, 22 (5), 2473–2478. 10.1116/1.1800351.

[ref30] Nunes R. W.; Martin J. R.; Johnson J. F. Influence of molecular weight and molecular weight distribution on mechanical properties of polymers. Polym. Eng. Sci. 1982, 22 (4), 205–228. 10.1002/pen.760220402.

[ref31] Perkins W. G. Polymer toughness and impact resistance. Polym. Eng. Sci. 1999, 39 (12), 2445–2460. 10.1002/pen.11632.

[ref32] Bhateja S. K. Uniaxial tensile creep behaviour of ultra high molecular weight linear polyethylene. Polymer 1981, 22 (1), 23–28. 10.1016/0032-3861(81)90071-9.

[ref33] Liu Y.; Klep V.; Zdyrko B.; Luzinov I. Synthesis of High-Density Grafted Polymer Layers with Thickness and Grafting Density Gradients. Langmuir 2005, 21 (25), 11806–11813. 10.1021/la051695q. 16316118

[ref34] Zhang J.; Kato K.; Uyama Y.; Ikada Y. Surface graft polymerization of glycidyl methacrylate onto polyethylene and the adhesion with epoxy resin. J. Polym. Sci., Part A: Polym. Chem. 1995, 33 (15), 2629–2638. 10.1002/pola.1995.080331509.

