# Supplementary material for: Enhancing the Kinetics of Vapor-based Polymerization by Pulsed Filament Approach
Source: Langmuir. 2024 Jul 16;40(30):15550–7. doi: 10.1021/acs.langmuir.4c01172 (PMC11295193; doi:10.1021/acs.langmuir.4c01172)
Supplement: Supplementary file 1 — la4c01172_si_001.pdf [file la4c01172_si_001.pdf]

Enhancing the kinetics of vapor-based polymerization by pulsed filament approach

Supporting Information

*Jie Guo, Ranjita K. Bose**

Department of Chemical Engineering, Product Technology, University of Groningen, Nijenborgh 4, 9747 AG, Groningen, the Netherlands

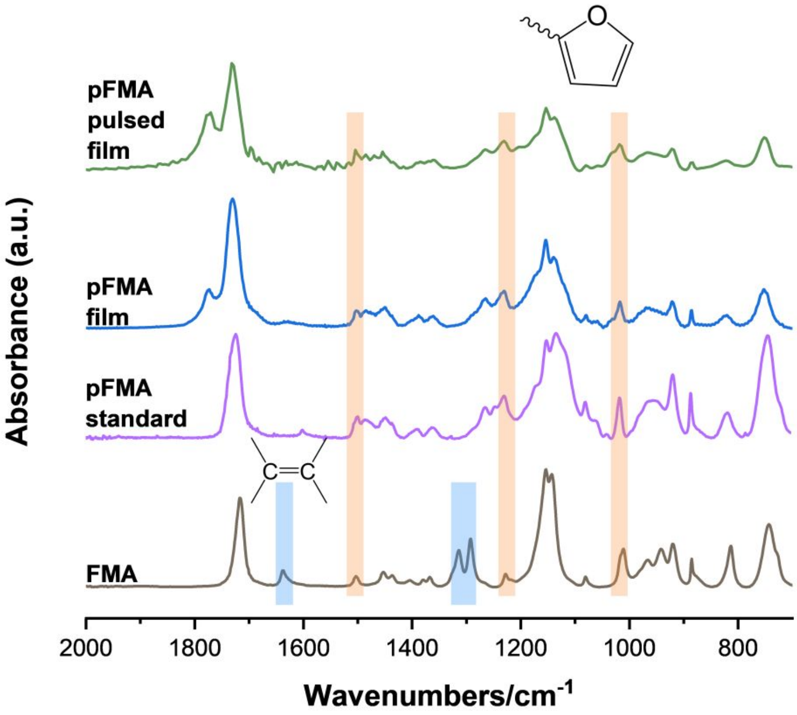

Figure S1 Infrared spectra of poly(furfuryl methacrylate), pFMA. The four curves represent three kinds of pFMA: pulsed iCVD pFMA film, continuous iCVD pFMA film, and standard pFMA synthesized by atom transfer radical polymerization and the monomer FMA.

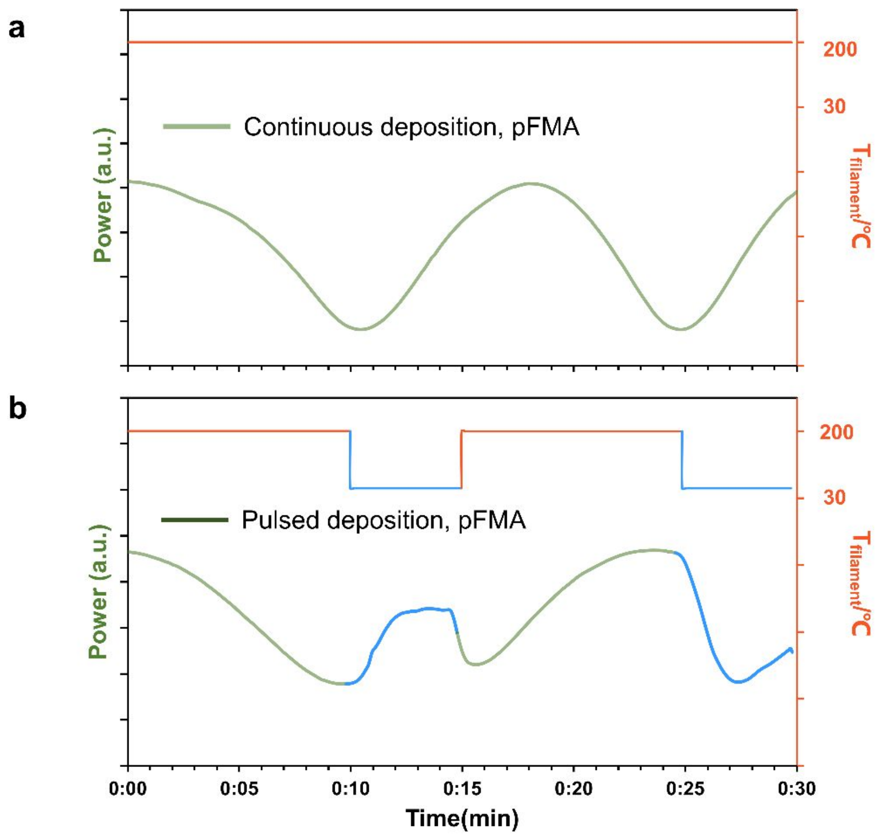

Figure S2 Laser interference pattern of the pFMA film on silicon substrates during initiated chemical vapor deposition: a, continuous deposition; b, pulsed deposition.

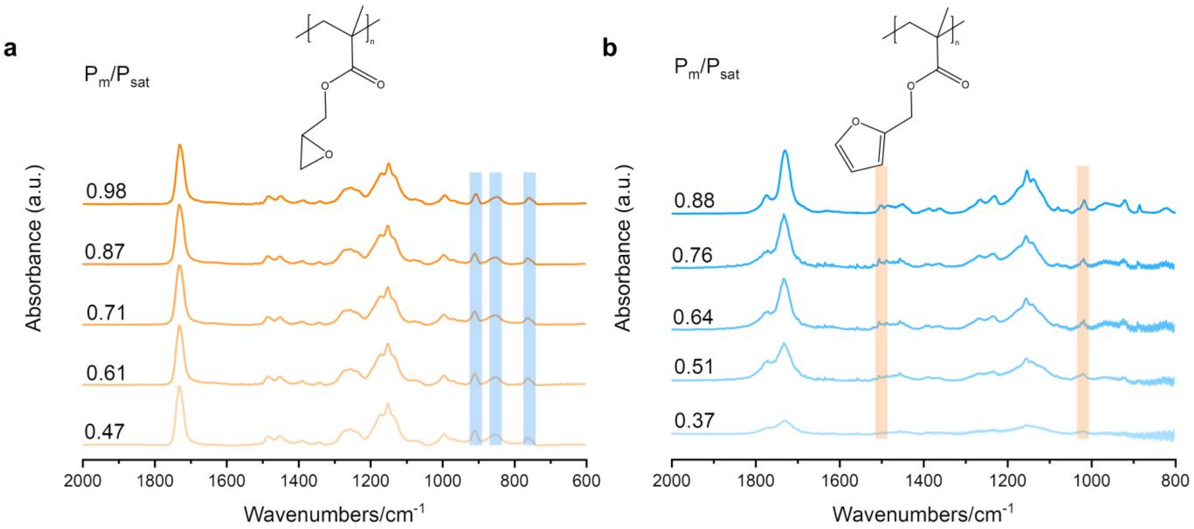

Figure S3 IR spectra of pGMA (left) and pFMA (right) at different $P_M/P_{M,sat}$ by controlling reactor pressures.

Table S1 Deposition parameters for pFMA. P_R, chamber pressure; F_M, flow rate of monomer; F_I, flow rate of initiator; F_{N2}, flow rate of nitrogen; T_S, substrate temperature; T_{fila}, filament temperature; $P_M/P_{M,sat}$, ratio of monomer partial pressure to the saturated pressure of monomer at T_S; $P_I/P_{I,sat}$, ratio of initiator partial pressure to the saturated pressure of initiator at T_S.

pFMA iCVD parameter	P_R/mTorr	F_M/sccm	F_I/sccm	F_{N2}/sccm	T_S/°C	T_{Fila}/°C	$P_M/P_{M,sat}$	$P_I/P_{I,sat}$
Continuous deposition	90	0.90	1.20	0.80	20	200	0.31	0.0015
	120	0.90	1.20	0.80	20	200	0.42	0.0020
	150	0.90	1.20	0.80	20	200	0.52	0.0025
	180	0.90	1.20	0.80	20	200	0.63	0.0030
	210	0.90	1.20	0.80	20	200	0.73	0.0035
Pulsed deposition	150	0.90	1.20	0.80	20	200/30	0.52	0.0025

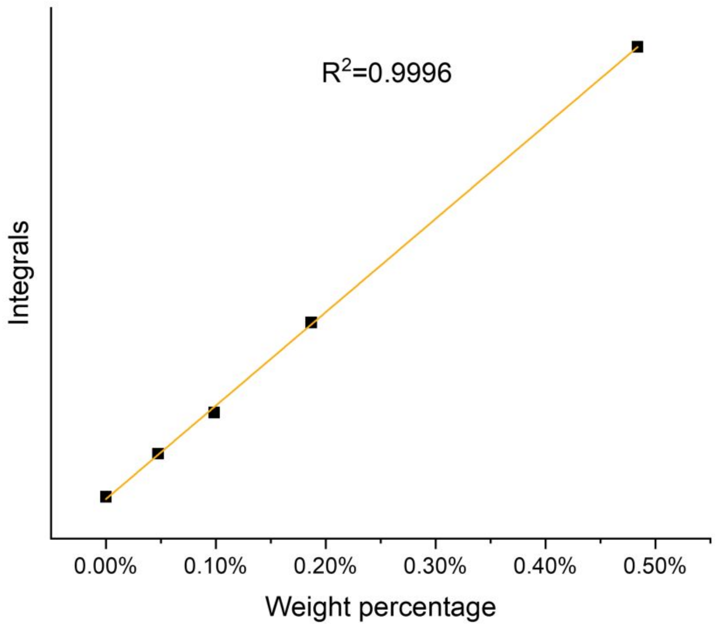

Figure S4 The standard calibration curve of the integrals of standard pGMA (Sigma Aldrich) dissolved in THF with different weight concentrations.
